# Brainstem Respiratory Center Dysfunction in Persons With Epilepsy: An fMRI Study

**DOI:** 10.1212/WNL.0000000000214603

**Published:** 2026-02-10

**Authors:** Carolina Ciumas, Romain Bouet, Andrea O. Rossetti, Jan Novy, Danielle Ibarrola, Sylvain Rheims, Philippe Ryvlin

**Affiliations:** 1 Department of Clinical Neurosciences, Lausanne University Hospital (CHUV) and University of Lausanne, Switzerland;; 2 Lyon Neuroscience Research Center (CRNL), Inserm U1028, CNRS UMR5292, and Lyon 1 University, France;; 3 Cermep - Imagerie du vivant, CNRS UAR3453, Lyon, France;; 4 Department of Functional Neurology and Epileptology, Hospices Civils de Lyon and Lyon 1 University, France; and; 5 member of ERN EpiCare.

## Abstract

**Background and Objectives:**

Peri-ictal apnea is common in persons with epilepsy (PWE) and may contribute to sudden unexpected death in epilepsy (SUDEP). It is not yet known whether brainstem respiratory centers in PWE respond differently to voluntary apnea. We addressed this issue using breath-holding (BH) functional MRI (fMRI).

**Methods:**

Adult PWE were recruited from a single outpatient clinic with the aim of achieving a comparable proportion of patients with and without generalized or focal-to-bilateral tonic-clonic seizures. Age-matched and sex-matched healthy controls were recruited through public advertisements. Exclusion criteria included physiologic and medical conditions known to alter respiration. All participants underwent fMRI while performing voluntary inspiratory and expiratory BH tasks. Respiratory rate, oxygen saturation, and end-tidal O_2_ and CO_2_ were recorded. Functional data were analyzed using a standard general linear model, as well as seed-to-voxel and ROI-to-ROI connectivity analyses targeting predefined brainstem regions of interest.

**Results:**

The study included 31 PWE (mean age 34.0 ± 11.6 years, 51.6% female) and 21 controls (mean age 32.8 ± 9.9, 52.4% female). At the group level, PWE had significantly lower brainstem activation than controls during both expiratory BH (Cohen *d* = 1.43, 95% CI 0.81–2.05, *p* = 0.005) and inspiratory BH (Cohen *d* = 1.31, 95% CI 0.70–1.92, *p* = 0.006). Decreased activations in PWE were observed in regions corresponding to the cuneiform nucleus during expiratory BH and the median raphe nucleus during the inspiratory BH. BH-triggered fMRI changes at the individual level showed a significant brainstem activation during expiratory BH in 61% of PWE vs 90% of controls. When comparing each participant with the control group, 35% of PWE demonstrated a significantly decreased brainstem activation, vs none of the controls. Intrinsic connectivity during self-paced breathing and BH showed reduced brainstem-cortical connectivity in PWE.

**Discussion:**

A significant proportion of PWE seem to suffer from interictal dysfunction of brainstem regions involved in respiratory control. These abnormalities could be detected at the individual level using a simple BH fMRI paradigm, suggesting potential for translation into a clinical biomarker. Future studies are needed to confirm these findings in larger populations and investigate their relation to SUDEP.

## Introduction

Most cases of sudden unexpected death in epilepsy (SUDEP) were found to primarily result from postictal apnea, after loss of physiologic breathing reflex.^1^ Accordingly, structural and biochemical abnormalities were observed in brainstem respiratory centers.^2,3^ Several functional MRI methods have been used to investigate brainstem response to experimental manipulations of respiration in persons with epilepsy (PWE).^4-6^ One such method, using iso-oxic hypercapnic breathing challenges, found increased brainstem BOLD responses in PWE compared with controls.^7^ Voluntary breath-holding (BH) functional MRI (fMRI) is another potent method to activate brainstem respiratory centers, which we recently calibrated in healthy individuals.^8^ BH induces a complex physiologic response combining hypoxia and hypercapnia that may reveal deficits in autonomic and chemosensory control in PWE. Given the lack of appropriate response to apnea observed during SUDEP, we hypothesized that some PWE might suffer from reduced brainstem fMRI activation in response to BH.

## Methods

### Participants

Participants included adult PWE from the outpatient epilepsy clinic of Lausanne University Hospital (Switzerland) and previously reported healthy controls.^8^ Recruitment of PWE aimed at achieving a comparable proportion of patients with and without a GTCS or a FBTCS during the past 12 months. Exclusion criteria were as follows: (1) ongoing or chronic medical conditions, other than epilepsy for PWE; (2) known respiratory disorders; (3) current use of medications unrelated to epilepsy treatment or hormonal contraception; (4) pregnancy; (5) permanent residence at altitude above 2000 meters; (6) engagement in regular breath-hold diving; and (7) any condition that contraindicates MRI scanning.

### Standard Protocol Approvals, Registrations, and Participant Consents

The Ethics Committee of the Canton de Vaud reviewed and approved this study protocol (approval number 2016–02057). Written informed consent was obtained.

### Physiologic Signals

Respiratory efforts were recorded using an MRI-compatible thoracic belt, and blood oxygenation was monitored through fingertip pulse oximetry. In addition, end-tidal oxygen and carbon dioxide levels were measured using a mouthpiece equipped with a cannula (Biopac Systems).

### Behavioral Experiment

The experiment is detailed elsewhere.^8^ Each participant completed 4 BH runs (2 inspiratory and 2 expiratory). Within each run, 6 BH blocks (≈20–40 seconds each) were performed. BH blocks were interleaved with 30 seconds of self-paced breathing, cued by a green dot, to allow recovery and return to baseline. Runs commenced with 90 seconds of self-paced breathing and concluded with 60 seconds of spontaneous breathing after the final (sixth) BH block. Task cues consisted of a 2-second yellow dot indicating preparation for BH, followed by a red dot signaling the onset and maintenance of BH. On resumption of breathing, participants pressed a response button, at which point the visual cue reverted to green (Presentation software, Neurobehavioral Systems) (eFigure 1).

### MRI Data Acquisition

Participants were scanned using a 3T MRI scanner (Magnetom Prisma, Siemens, Germany). The structural imaging protocol comprised high-resolution anatomical acquisition (sagittal MPRAGE sequence; slice tilt = 8°, echo time = 2.67 ms, repetition time = 3.50 seconds, inversion time = 1,000 ms, matrix = 256, field of view = 224 mm, slice thickness = 0.9 mm, voxel dimensions = 0.9 × 0.9 × 0.9 mm), followed by a time-of-flight (TOF) angiographic sequence (slice tilt = 18°, echo time = 3.43 ms, repetition time = 0.21 seconds, slice thickness = 0.6 mm, GRAPPA acceleration factor = 2). TOF and T1 images were used to confirm the anatomical localization of functional activations relative to brainstem nuclei and to rule out vascular confounding factors.

Functional scans used T2*-weighted 2D EPI (TR = 2.52 seconds, TE = 30 ms, flip angle = 80°, slice thickness = 2.5 mm, spacing = 3.125 mm, GRAPPA = 2, bandwidth = 2,470 Hz/pixel). The in-plane resolution was 2.5 × 2.5 mm^2^ (matrix 84 × 88; FoV≈210 × 220 mm). The phase-encoding direction was COL, corresponding to left-right for the coronal-oblique prescription (≈40°tilt relative to AC–PC). No explicit distortion correction (field-map or reverse-phase encoding unwarping) was performed; susceptibility effects were mitigated by the slice prescription and the high readout bandwidth. Sequences were optimized to cover both the brain and brainstem and validated in previous studies for reliable detection of brainstem BOLD signals.^9-11^

### MRI Data Preprocessing

MRI data were analyzed with MATLAB7.6 and SPM12 in the native space before being normalized to MNI space. To achieve MRI signal equilibrium, the first 4 volumes were removed. The images were then realigned to the mean functional image, which was co-registered with the anatomical image. Volumes exceeding 0.5 mm of framewise displacement or 3 standard deviations in the global signal (ART-toolbox) were excluded. Tissue segmentation was performed using SPM12. fMRI volumes were then resliced in MNI space to voxels of 2 × 2 × 2 mm and smoothed with a 4-mm full-width half-maximum kernel.

fMRI studies of the human brainstem have posed significant methodological challenges.^12,13^ The BH maneuver leads to distinct and rapid BOLD-like signal fluctuations, which require appropriate filtering.^14,15^ Moreover, BH modifies O_2_ and CO_2_ levels, leading to slight hypoxia and elevated hypercapnia, both of which influence the BOLD signal. Furthermore, changes in blood pressure and cerebral blood volume with every heartbeat generate motion-related artifacts connected to cardiac activity.^16^ To mitigate these confounding factors, the end-tidal CO_2_ traces were modeled using a hemodynamic response function and regressed out of the BOLD signal on a voxel-by-voxel basis. End-tidal CO_2_ was sampled using a mouthpiece and cannula (5.3-m tubing, shortened from the standard 10 m). The sampling delay introduced by the tubing was measured and corrected in AcqKnowledge software (Biopac) before HRF convolution and regression.

End-tidal CO_2_ values were extracted at the end of each expiration, and a linear interpolation was applied to align with the repetition interval (2520 ms). During BH with sustained apnea from onset of the task, no exhale and thus no end-tidal CO_2_ is available during the entire BH period. We thus evaluated the impact of BH on end-tidal CO_2_ by subtracting the values obtained for the last exhale before BH and the first one after BH.^17^

### Breath-Hold Timing and Data Rejection

The timing of each BH was established using automatic recordings from the Presentation program and by analysis of recorded respiratory movements with in-house MATLAB-based software. The different sources of data produced consistent results, with occasional minor delays or premature termination relative to the cue. We defined apnea during BH as an airflow cessation for more than 10 seconds. Airflow cessation was controlled by inspecting respiratory movements and variations of end-tidal CO_2_ and O_2_ during BH. BH blocks with detectable change in end-tidal gas concentrations or thoracic movements indicating lack of sustained BH were eliminated. The self-paced breathing periods at the start and end of each fMRI run were used as the baseline because they were distant from any apnea and thus unaffected by postapnea recovery. Owing to the delayed and spatially variable BOLD response induced by BH,^18^ intertrial free breathing was excluded.

### Behavioral and Physiologic Validation

We evaluated the agreement between manually corrected and automatically recorded apnea duration using correlation analysis. Consistent with previous reports, self-reported apnea durations highly correlated with those derived from respiratory recordings (R = 0.963 for expiratory BH; R = 0.954 for inspiratory BH).^8^ The variation in oxygen desaturation (SpO_2_ drop) between inspiratory and expiratory breath-holds was assessed with a Mann-Whitney U test.

### Definition of Region of Interest (ROI)

We selected 13 brainstem ROIs from the Brainstem Navigator probabilistic atlas.^19-22^ These included the left and right medial parabrachial nuclei, the left and right lateral parabrachial nuclei, locus coeruleus, inferior medullary reticular formations, and superior medullary reticular formations, as well as the median raphe, dorsal raphe, and periaqueductal gray.

### Statistical Analyses

#### Behavioral and Physiologic Analyses

Nonparametric tests were used to compare behavioral and physiologic parameters. Within-group comparisons were assessed using the Wilcoxon signed-rank test; between-group comparisons were assessed using the Mann-Whitney *U* test. Correlations were evaluated using Spearman rho.

#### Task-Based BOLD Activation Analyses

Voxel-wise BOLD activation was analyzed using general linear models (GLMs) in SPM12. First-level models incorporated condition regressors for expiratory and inspiratory breath-holds, 6 motion parameters, end-tidal CO_2_, respiratory and cardiac signals,^9,14,23^ and mean white matter signals. Individual-level contrasts included the following: (1) expiratory BH vs baseline; (2) inspiratory BH vs baseline; (3) expiratory BH vs inspiratory BH; (4) inspiratory BH vs expiratory BH; (5) baseline vs inspiratory BH; (6) baseline vs expiratory BH.

Between-group contrasts were computed for each condition. Second-level random-effects models included age, sex, BMI (used as a surrogate of potentially undiagnosed sleep obstructive apnea syndrome), and framewise displacement as covariates. Analyses were restricted to a brainstem mask derived from the Hammersmith atlas (n30r83). Group-level statistical maps were thresholded at *p* < 0.05, family-wise error-corrected (peak and cluster levels). We performed sensitivity analyses excluding patients receiving medication that could affect respiration, such as topiramate or zonisamide.

Individual BOLD activation maps were contrasted against group-level maps from controls (with the tested control participant excluded when applicable) to identify participant-specific deviations from normative patterns. The location of statistically significant clusters within the brainstem was determined using WIKIBrainStem^24^ and Brainstem Navigator atlas toolkit (available at NITRC), which provides probabilistic in vivo labels of human brainstem nuclei derived from 7T MRI.^19-22^

We investigated correlations between the BOLD signal during expiratory and inspiratory BH tasks and the following clinical variables: sex, time since last seizure, time since last GTCS/FBTCS, total number of seizures per year, and duration of epilepsy. We also assessed correlations with the BH duration and SpO_2_ variations.

#### Functional Connectivity Analyses

The functional connectivity analyses were performed using the CONN toolbox (v22a).^25^ First-level SPM models, including regressors for inspiratory and expiratory BH, motion parameters, respiratory and cardiac traces, and mean WM signals, were imported into CONN for connectivity analyses. During CONN denoising, inspiratory and expiratory BH regressors were explicitly included as nuisance covariates, alongside WM/CSF signals and realignment parameters. After regression (RegBP), a temporal band-pass filter (0.008–0.09 Hz) was applied to the residual BOLD time series. Thus, connectivity was estimated using task-residualized signals. It should be noted that WM/CSF and motion parameters were included both at the first level and in CONN's denoising process, providing a conservative double regression. The analyses encompassed both seed-to-voxel (SBC) and ROI-to-ROI (RRC) approaches. Predefined seed ROIs were obtained from Brainstem Navigator. Correlation coefficients between regions of interest (RRC) and the BOLD time series of a specific ROI, along with the BOLD time series of each individual voxel (SBC), were computed. Cluster-level inferences were based on Gaussian Random Field theory.^26^ Results were thresholded using a combination of a cluster-forming voxel-level threshold of *p* < 0.001 and a cluster-size threshold of *p* < 0.05, FWE-corrected.^27^ Second-level SBC results were thresholded (voxel-level *p* < 0.001 uncorrected, cluster-level *p* < 0.05 FDR-corrected).

### Data Availability

Data published in this article are available on request from the corresponding authors.

## Results

### Participant Characteristics

A total of 56 participants were enrolled, including 35 PWE and 21 age-matched and sex-matched healthy controls. Four patients did not complete the experiment and were excluded. Two of them did not finish the 4 fMRI runs while the other 2 quit the experiment after the anatomical MRI because of anxiety and discomfort from using a mouthpiece for breathing (flow diagram in eFigure 2). The remaining 31 patients had a mean age SD of 34.0 ± 11.6 years, with 51.6% female and a mean BMI of 23.9 ± 4.3 kg/m^2^, while the 21 controls had a mean age ± SD of 32.8 ± 9.9 years, with 52.4% female and a mean BMI of 22.5 ± 3.4 kg/m^2^.

Clinical characteristics of PWE are presented in Table 1, detailed in eTable 1. Epilepsy was classified as idiopathic generalized epilepsy in 19 patients (61%) and focal in 12 (39%), reflecting our explicit recruitment bias to ensure a comparable proportion of patients with and without a GTCS or a FBTCS during the past year. Accordingly, 48% of PWE have had such a seizure during the past year. Seven patients did not suffer any seizure during that period, whereas the other 24 had a median number of 4 seizures within the past 12 months, with a median delay of one month between the last known seizure and the MRI study. The shortest delay was one day for strictly focal seizures and 4 days for GTCS/FBTCS.

**Table 1:** Demographic Data

Variables	Values
No. of participants	31
Sex (F/M)	16/15
Age range (y)	15–65
Median epilepsy duration (y)	5–10 y: 9 15–20 y: 8 >20 y: 6 <5 y: 5 10–15 y: 3
Family history of epilepsy	8/31
MRI abnormalities	6/31
FBTCS/GTCS in past year > 0	20
Monotherapy/polytherapy/none	16/14/1

### Behavioral Response

The mean (±SD) duration of apnea was longer for inspiratory BH than for expiratory BH in both PWE and healthy controls (32.1 ± 13.6 seconds vs 24.9 ± 10.7 seconds in PWE (*p* = 0.024); 46.1 ± 19.2 seconds vs 28.9 ± 7.9 seconds in controls (*p* < 0.001)). The mean duration of inspiratory BH was significantly shorter in PWE than in healthy controls (*p* = 0.003), but not that of expiratory BH (*p* = 0.147). The decrease in SpO_2_ during expiratory BH was comparable between groups (−2.2% ± 1.5 in PWE, −2.7% ± 2.2 in controls), but significantly greater in PWE (−1.8% ± 0.8) than in healthy controls (0.9% ± 0.8) during inspiratory BH (*p* < 0.001). The increase in ETCO_2_ during expiratory and inspiratory BH was comparable between groups and conditions (Table 2).

**Table 2:** Behavioral Data

Condition	Patients (N = 31)	Controls (N = 21)	Between-group comparison
	Mean ± SD (range)	Mean ± SD (range)	*p* Value
Inspiratory BH duration	**32.1 ± 13.6 (12.2–62.5)** ^a^	**46.1 ± 19.2 (24.6–98.1)** ^b^	0.003
Expiratory BH duration	**24.9 ± 10.7 (11.2–54.0)**	**28.9 ± 7.9 (17.5–52.0)**	NS
Inspiratory BH SpO_2_ drop	1.8 ± 0.8 (0.7–4.2)	**0.9 ± 0.8 (0.0–2.6)** ^c^	<0.001
Expiratory BH SpO_2_ drop	2.2 ± 1.5 (0.7–6.7)	**2.7 ± 2.2 (0.1–7.7)**	NS

Abbreviations: BH = breath-holding; NS = nonsignificant.

Numbers in bold indicate significant difference between the 2 conditions within the group.

a *p* = 0.024.

b *p* < 0.001.

c *p* = 0.001.

### fMRI Findings

#### Within-Group Analysis

**Figure 1:**
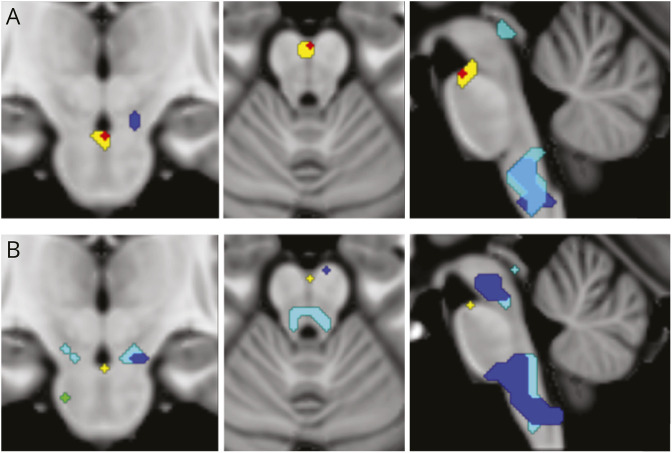
Within-Group Analyses The upper row shows the control group, and the lower row shows the patient group. (A) Controls. Activation and deactivation patterns during BH are shown relative to baseline. Activations appear in red for inspiratory BH vs baseline and in yellow for expiratory BH vs baseline. Deactivations appear in dark blue for inspiratory BH < baseline and in cyan for expiratory BH < baseline. Negative BOLD clusters were detected in the medulla (superior and inferior medullary reticular formations, raphe magnus, obscurus, and pallidus) and in the periaqueductal gray during both inspiratory and expiratory BH tasks. During expiratory BH, deactivation extended further into the dorsal raphe and cuneiform nuclei. No significant clusters were found for the direct comparison of inspiratory and expiratory BH tasks. (B) Patients. Same contrasts as in controls. No significant activation was observed for inspiratory BH > baseline (red cluster absent). A significant cluster was identified for expiratory BH > inspiratory BH (green, coronal slice). BH = breath-holding.

PWE demonstrated a single brainstem cluster of BOLD activation during expiratory BH over a region encompassing the ventral mesencephalo-pontine area that includes the mesencephalic reticular formation^24^ and parabrachial complex, overlapping with the region often termed the Kölliker-Fuse in physiologic studies (x = 0, y = −19, z = −22, cluster size 0.128 cm^3^) (Figure 1). By contrast, no significant activation was observed during inspiratory BH. When directly comparing both conditions, a significant cluster was observed at the border between the lateral pons (x = 6, y = −25, z = −46, cluster size 0.64 cm^3^), indicating greater BOLD response for expiratory BH than for inspiratory BH (visible in the coronal view of the second row in Figure 1B). Deactivation clusters during both conditions were observed in the medulla (superior and inferior medullary reticular formations, raphe magnus, obscurus, and pallidus), as well as in the periaqueductal gray. During expiratory BH, additional deactivation extended into the dorsal raphe and cuneiform nucleus (Figure 1, A and B). These deactivations did not significantly differ between expiratory and inspiratory BH. Excluding patients on topiramate or zonisamide had minimal effect on group-level brainstem activation. Expiratory BH results remained unchanged while task-related deactivations showed a slight decrease in both conditions.

During expiratory BH, 2 activation clusters were observed in the control group: a small cluster in the left pontine nuclei (Duvernoy Brainstem Atlas)^28^ (x = 12, y = −22, z = −31, cluster size 0.128 cm^3^) and a larger cluster in the same brainstem region activated in PWE, but with greater extent in the control group (cluster size = 0.448 cm^3^ vs 0.128 cm^3^). The same region was active during inspiratory BH, with no significant difference between conditions (Figure 1). Furthermore, deactivations during both tasks were much lower than those seen in PWE.

#### Between-Group Comparison

**Figure 2:**
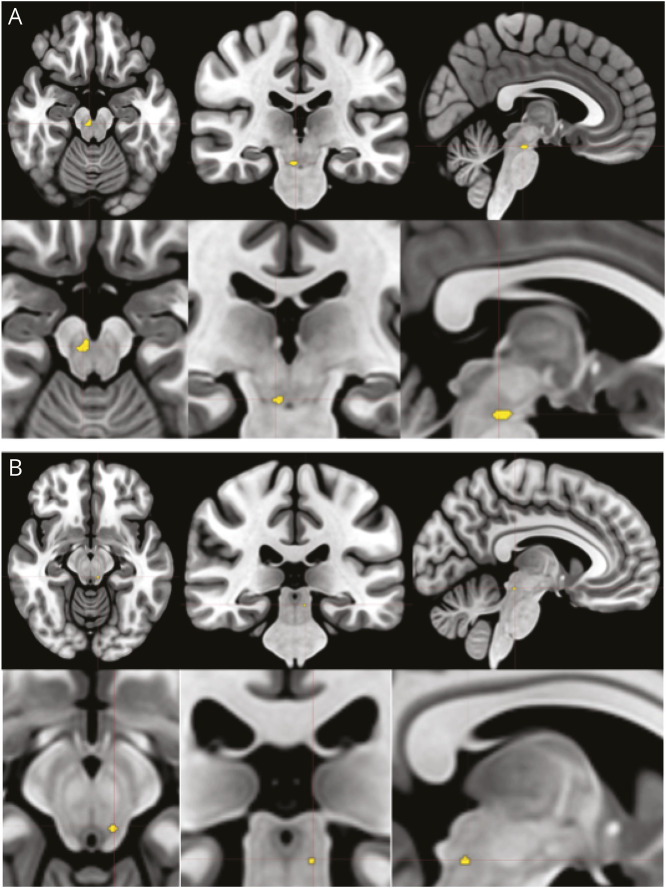
Between-Group Analyses Between-group differences during BH. (A) Expiratory BH: reduced activation in PWE was localized to a ventro-caudal mesencephalic-ponto-bulbar region encompassing the pedunculopontine and adjacent cuneiform nuclei. (B) Inspiratory BH: reduced activation in PWE was localized to the median raphe nucleus along the dorsal midline. BH = breath-holding; PWE = persons with epilepsy.

When directly comparing the 2 groups, significant differences were observed for both expiratory and inspiratory BH tasks. During expiratory BH, PWE showed less activation than controls in a ventro-caudal mesencephalo-pontine region, which encompasses the pedunculopontine and cuneiform nuclei (as defined in Brainstem Navigator) (x = −6, y = −31, z = −13, cluster size 0.64 cm^3^) (Cohen *d* = 1.43, 95% CI 0.81–2.05, *p* = 0.005), whereas during inspiratory BH, PWE showed less activation than controls over a region encompassing the median raphe nucleus (x = 3, y = −25, z = −19, cluster size 0.192 cm^3^) (Cohen *d* = 1.31, 95% CI 0.70–1.92, *p* = 0.006) (Figure 2). Integrating apnea duration in the GLM model did not change these findings.

#### Individual Imaging Analysis

Among the control group, 19 of 21 (90%) showed significant brainstem activation during expiratory BH and 13 of 21 (62%) during inspiratory BH (eTable 2). Of the PWE, 20 of 31 (64%) exhibited significant activation (61% expiratory and 29% inspiratory; eTable 2 and eFigure 3), with no significant difference between the 2 groups (*p* = 0.07). Deactivations occurred in 81% of PWE and 86% of controls during expiratory BH and in 74% and 81%, respectively, during inspiratory BH. These were predominantly located in the medulla, but often extended to the pons (detailed in the eMethods; eTable 3 provides the distribution of individual brainstem BOLD responses as a function of apnea duration).

#### Individual vs Control Group Comparison

Comparison of each of the 31 PWE with the control group showed significantly decreased activation in 11 PWE (35%) during the expiratory BH task. This reduced activation was located in the mesencephalon in 6 patients (19%), the pons in 3 (9%), and the medulla in 5 (16%). Six PWE (19%) demonstrated significantly reduced brainstem activation during inspiratory BH, over the midbrain or medulla in 3 patients (9%) and in the pons in 2 (6%). None of the controls showed significant difference when compared with the control group.

#### Associations With Clinical Variables in PWE

Only one of the investigated associations was statistically significant, that is, a positive correlation between the total number of seizures per year and the BOLD activation during inspiratory BH, with a cluster located over the medullar nuclei (x = 3, y = −35, z = −55, eFigure 4).

#### Functional Connectivity

**Figure 3:**
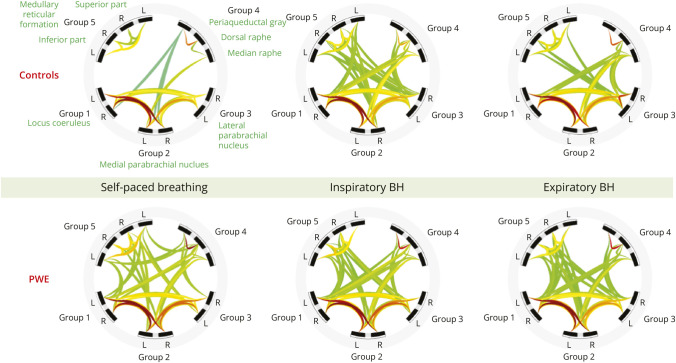
Brainstem ROI-to-ROI Functional Connectivity at Rest and During BH in Controls and PWE No significant differences were found between groups. Similar patterns were observed in both groups, with strongest connectivity between parabrachial nuclei and locus coeruleus. Connectivity increased during BH, especially between locus coeruleus and inferior medullary reticular formation. Strength of connectivity is represented across a color spectrum, with red representing larger T-values and stronger connectivity and green representing lighter T-values. BH = breath-holding; PWE = persons with epilepsy.

Brainstem ROI-to-ROI analysis did not disclose significant difference in functional connectivity between PWE and controls. In both populations, the most prominent ROI-to-ROI functional connectivity was observed between the medial parabrachial nucleus on the one hand and the locus coeruleus and lateral parabrachial nucleus on the other hand. This connectivity pattern was similar at normal breathing and during BH. Other less prominent functional connectivity involved the same 3 regions as above, as well as the dorsal raphe, median raphe, the medullary reticular formation, the periaqueductal gray, and the superior and inferior reticular formation. Except for the raphe nuclei, these ROIs showed an increased functional connectivity during BH. The largest increase was observed in the connection between the locus coeruleus and the inferior medullary reticular formation, particularly during inspiratory BH (Figure 3).

In normal breathing state analyses, seed-based functional connectivity showed a reduced connectivity in PWE compared with controls for the dorsal raphe (peak MNI: x = −42, y = 35, z = 17; cluster-level FWE *p* = 0.020) and the medullary inferior reticular formation (x = −24, y = −61, z = 47; cluster-level FWE *p* = 0.006). The dorsal raphe showed reduced connectivity with several left frontal regions (precentral, inferior, and middle frontal gyri) while the medullary inferior reticular formation showed reduced connectivity with the left lateral occipital cortex, superior parietal lobule, and frontal pole.

**Figure 4:**
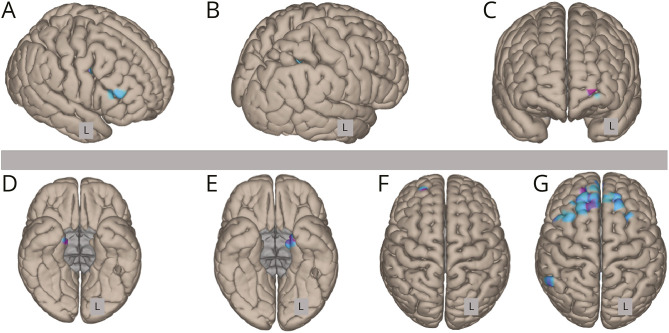
Seed-to-Voxel Connectivity Analyses Patients exhibited a significant reduction in connectivity compared with controls. Panels A–C (top row) correspond to the self-paced breathing condition, and panels D–G (bottom row) correspond to the expiratory BH condition. “L” indicates the left hemisphere. No clusters were identified during inspiratory BH. The following ROIs showed reduced connectivity: A, dorsal raphe; B, left inferior medullary reticular formation; C, right inferior medullary reticular formation; D, right medial parabrachial nucleus; E, locus coeruleus; F, left lateral parabrachial nucleus; G, median raphe. BH = breath-holding.

PWE also showed significant reduction in functional connectivity compared with controls during expiratory BH. More specifically, the locus coeruleus showed reduced connectivity with the left parahippocampal gyrus, hippocampus, and amygdala (x = −30, y = −16, z = −31; cluster-level FWE *p* = 0.002), while the medial parabrachial nucleus showed a reduced connectivity with the same set of regions but on the right side (x = 22, y = −18, z = −14; cluster-level FWE *p* = 0.006). The lateral parabrachial nucleus also showed a reduced connectivity with the right frontal pole (x = 3, y = 69, z = 6; cluster-level FWE *p* = 0.039). Finally, the median raphe showed a reduced connectivity with many frontal and parietal regions on both sides (right superior frontal gyrus, bilateral frontal pole, superior and middle frontal gyri, paracingulate and posterior cingulate gyri, and right angular gyrus and precuneus) (x = 0, y = −31, z = −19; cluster-level FWE *p* < 0.001) (Figure 4). No difference in functional connectivity was observed between the 2 groups during inspiratory BH.

## Discussion

This study showed differences between PWE and controls in fMRI brainstem response to BH, suggesting epilepsy-related changes in the activity of some respiration-related centers, part of which could be detected at the individual patient level.

All participants except one control and 2 patients could perform an appropriate BH task, indicating that this simple task can be managed by most PWE. We tested both inspiratory and expiratory BH tasks because these engage distinct respiratory control mechanisms and found that expiratory BH was more suitable than inspiratory BH for demonstrating epilepsy-related fMRI abnormalities. Only 2 6-minute runs are needed to obtain informative expiratory BH fMRI, suggesting its potential as a biomarker if later proved clinically relevant. Self-timed BH was selected to maximize the feasibility and comfort of the task for PWE. Yet, externally cued BH may reduce interparticipant variability in future work.

We observed BH-triggered fMRI brainstem activations in both groups, which proved significant at the stringent FWE-corrected level. We believe that these activations truly reflect changes in neural activity of brainstem nuclei, given that our model controls for the potential impact of respiratory and cardiac movements–related artifacts. Significant clusters only involved a few voxels, a finding consistent with the small size of most brainstem nuclei. End-tidal gas traces were corrected for the sampling delay introduced by the tubing. Any residual temporal mismatch is expected to have a negligible impact on the results.

The brainstem region that was primarily activated by BH encompasses the mesencephalic median reticular nuclei,^29^ which receive input from pontine and medullary centers and integrate this information to control timing and intensity of expiration.^30^ These nuclei are thus likely activated by a prolonged BH, disrupting the physiologic timing of expiration. Two other fMRI studies have investigated the response of brainstem respiratory centers to BH or CO_2_ challenge.^31,32^ They reported a significant increase in BOLD signal in the dorsal pons and in the medulla, but not over the mesencephalic median reticular nuclei.^31,32^ Discrepancies with our findings may be due to differences in experimental designs (BH vs CO_2_ challenge, longer BH duration in our study resulting in higher ETCO_2_ increase), populations (with larger sample size in our study), and pre/postprocessing methods.

We also observed BH-induced fMRI deactivation over the medulla and mesencephalic periaqueductal gray matter in both PWE and controls. Accordingly, 2 other series have reported fMRI deactivation during expiratory BH, either over the medulla^31^ or the periaqueductal gray matter.^33^ A more complex pattern was observed over the medulla after a hypercapnic challenge, with a transient 10-second BOLD signal increase followed by a decrease.^34^ This deactivation might reflect a post-BH activation triggered by the urge to breathe and increased CO_2_ levels. Another explanation could be a top-down inhibition of brainstem respiratory centers to achieve voluntary BH. Alternatively, such deactivations may reflect reductions in cerebrovascular reactivity or a vascular steal phenomenon during hypercapnic conditions. While these deactivations are consistent with brainstem nuclei involved in respiratory and autonomic control, caution should be exercised in their interpretation, given the known limits of spatial resolution and susceptibility artifacts in this region.

BH-triggered fMRI activations differed between expiratory and inspiratory BH tasks and between groups. PWE showed less activation than controls during both tasks. During BH challenges, PWE exhibited reduced activation in key ponto-mesencephalic nuclei involved in respiratory control and arousal. During expiratory BH, the response was attenuated in the dorsal region encompassing the cuneiform nucleus, which is part of the brainstem survival network and is connected to the periaqueductal gray.^35^ This nucleus also projects to the preBötzinger complex,^36^ which modulates arousal and respiratory drive. During inspiratory BH, activation in the median raphe nucleus (MnR) was also markedly reduced in people with epilepsy. The MnR is a central serotonergic structure involved in chemosensory and cardiorespiratory regulation,^37^ particularly under hypercapnic stress.^38^ Reduced MnR and cuneiform responses in PWE may indicate impaired brainstem integration of respiratory and autonomic signals, which could lead to peri-ictal autonomic instability and an increased risk of SUDEP. By contrast, CO_2_ challenge fMRI showed greater brainstem BOLD activations in PWE compared with controls over the dorsal raphe nucleus. This discrepancy might reflect differences in the CO_2_ levels associated with the 2 methods.^7^ BH causes a gradual increase in CO_2_ and decrease in O_2_,^39^ whereas CO_2_ administration causes a rapid increase in CO_2_ with minimal changes in O_2_.^40^ Furthermore, the voluntary control of respiration during BH is likely to stimulate respiratory centers, regardless of CO_2_ and O_2_ levels.

Significant BOLD responses to BH were observed at the individual level, with all but 2 of the controls showing brainstem activation during expiratory BH. By contrast, only 61% of PWE had significant activations and 35% showed a significantly abnormal fMRI response when compared with the control group. These abnormalities could reflect seizure-related lesional or functional changes in respiratory centers, effects of antiseizure medications, or a decreased neurovascular coupling.

Functional connectivity between brainstem ROIs showed an interesting pattern of increased connectivity during BH in both PWE and controls. This increased connectivity involved brainstem regions known to be highly interconnected and to modulate each other's activity, including the locus coeruleus and the parabrachial nuclei.^41,42^ Compared with controls, PWE showed decreased SBC-based functional connectivity between several brainstem nuclei (dorsal raphe, medullary inferior reticular formation, locus coeruleus, medial parabrachial nucleus) and various cortical brain regions, suggesting impaired brainstem-cortical integration in PWE. These findings were observed both at rest and during expiratory BH but proved more robust during BH. By contrast, a previous fMRI study using CO_2_ challenge reported increased connectivity in PWE compared with controls.^7^ In another resting-state study that compared PWE at high and low risk of SUDEP, the former showed heightened functional connectivity within a network that included the right side of the brainstem and several frontal cortical and subcortical regions, and reduced functional connectivity within a network that included the brainstem, the amygdala, the thalamus, and the putamen.^43^ Comparing the abovementioned results with our findings is challenging because of differences in methodology, but all studies point to the possibility of an altered regulation of the brainstem respiratory centers by cortico-subcortical networks in PWE.

Several limitations need to be emphasized, particularly the challenges of ensuring the lack of vascular artifacts and precise localization of fMRI findings within the brainstem. The small size and close physical proximity of respiratory centers make it difficult to ascribe specific centers to each observed fMRI activation and abnormality.^44^ We attempted to minimize these issues by using a combination of methods, including data analysis in native space overlaid on individual T1 images and TOF angiography to identify vessels, but acknowledge that uncertainty remains. Although OSA was an exclusion criterion, the presence of undiagnosed cases may have contributed to the fMRI abnormalities observed in PWE. We partly addressed this issue by integrating BMI as a covariate in our fMRI models. Our modeling strategy relied on HRF-convolved end-tidal CO_2_ regressors and voxel-wise nuisance regression, which may also affect sensitivity to subtle activations. This should be considered when comparing protocols. Our use of standard preprocessing protocols (e.g., whole-brain normalization at standard resolution) may limit precise brainstem localization. Future studies could improve on this by using higher resolution imaging and brainstem-specific normalization approaches. Notably, specialized processing could be applied to whole-brain acquisitions by isolating the brainstem for dedicated analysis—an approach likely to improve localization of brainstem fMRI activation. Our protocol did not include explicit distortion correction, but we minimized susceptibility artifacts using coronal-oblique slices and high bandwidth, as is standard in brainstem fMRI. A limitation of our preprocessing strategy is the inclusion of the mean WM signal as a nuisance regressor. Because SPM segmentation classifies most of the brainstem as WM, this step may remove variance related to genuine brainstem activations, leading to an underestimation of their magnitude. However, our significant findings emerged despite this conservative denoising strategy, reinforcing the robustness of the observed effects. Finally, the relatively small size of our cohorts and the heterogeneity of our patient population may have reduced the statistical power of our analyses, contributing to some of our negative findings.

It is important to note that our study was not designed to assess SUDEP risk, and no SUDEP-related outcome data are available in this cohort. Consequently, the current results should not be interpreted in a predictive or biomarker framework. Instead, they provide mechanistic insight into the neural correlates of respiratory dysfunction in epilepsy, particularly involving pontine and medullary nuclei implicated in ventilatory and autonomic control. BH-fMRI provides a noninvasive approach to investigating these physiologic processes in vivo, contributing to a better understanding of the pathways that may underlie vulnerability to peri-ictal respiratory compromise.

Overall, this study provides evidence that the response of brainstem respiration-related centers to voluntary apnea, as well as their functional connectivity, is altered in a significant proportion of PWE. These abnormalities were not associated with a history of GTCS/FBCTS, raising the interesting possibility that they could represent a risk factor of SUDEP independent from the role of GTCS/FBCTS. Future studies at a larger scale are warranted to test whether BH-fMRI or resting-state functional connectivity could prove clinically relevant, particularly to predict the risk of SUDEP.

## Glossary

BH: breath-holding
fMRI: functional MRI
GLM: general linear model
PWE: persons with epilepsy
ROI: regions of interest
SUDEP: sudden unexpected death in epilepsy
TOF: time-of-flight
